# Screening of Parkinson’s Disease Using Geometric Features Extracted from Spiral Drawings

**DOI:** 10.3390/brainsci11101297

**Published:** 2021-09-29

**Authors:** Jay Chandra, Siva Muthupalaniappan, Zisheng Shang, Richard Deng, Raymond Lin, Irina Tolkova, Dignity Butts, Daniel Sul, Sammer Marzouk, Soham Bose, Alexander Chen, Anushka Bhaskar, Sreekar Mantena, Daniel Z. Press

**Affiliations:** 1 Harvard College, Harvard University, Cambridge, MA 02138, USA; siva_muthupalaniappan@college.harvard.edu (S.M.); raymondlin@college.harvard.edu (R.L.); dignitybutts@college.harvard.edu (D.B.); smarzouk@college.harvard.edu (S.M.); sohambose@college.harvard.edu (S.B.); ajchen@college.harvard.edu (A.C.); anushkabhaskar@college.harvard.edu (A.B.); smantena@college.harvard.edu (S.M.); 2 Global Alliance for Medical Innovation, Cambridge, MA 02138, USA; jason.shang@duke.edu (Z.S.); richard.deng@duke.edu (R.D.); dds41@duke.edu (D.S.); 3 Trinity College of Arts and Sciences, Duke University, Durham, NC 27708, USA; 4 Pratt School of Engineering, Duke University, Durham, NC 27708, USA; 5 School of Engineering and Applied Sciences, Harvard University, Cambridge, MA 02138, USA; itolkova@g.harvard.edu; 6 Cognitive Neurology Unit, Beth Israel Deaconess Medical Center, Boston, MA 02215, USA; dpress@bidmc.harvard.edu; 7 Harvard Medical School, Harvard University, Boston, MA 02115, USA

**Keywords:** Parkinson’s Disease, biomarker, Archimedean spiral, disease screening, digitized drawing, machine learning, feature extraction

## Abstract

Conventional means of Parkinson’s Disease (PD) screening rely on qualitative tests typically administered by trained neurologists. Tablet technologies that enable data collection during handwriting and drawing tasks may provide low-cost, portable, and instantaneous quantitative methods for high-throughput PD screening. However, past efforts to use data from tablet-based drawing processes to distinguish between PD and control populations have demonstrated only moderate classification ability. Focusing on digitized drawings of Archimedean spirals, the present study utilized data from the open-access ParkinsonHW dataset to improve existing PD drawing diagnostic pipelines. Random forest classifiers were constructed using previously documented features and highly-predictive, newly-proposed features that leverage the many unique mathematical characteristics of the Archimedean spiral. This approach yielded an AUC of 0.999 on the particular dataset we tested on, and more importantly identified interpretable features with good promise for generalization across diverse patient cohorts. It demonstrated the potency of mathematical relationships inherent to the drawing shape and the usefulness of sparse feature sets and simple models, which further enhance interpretability, in the face of limited sample size. The results of this study also inform suggestions for future drawing task design and data analytics (feature extraction, shape selection, task diversity, drawing templates, and data sharing).

## 1. Introduction

### 1.1. Parkinson’s Disease Symptoms and Standard Diagnosis Methods

The diagnosis of Parkinson’s Disease (PD) has proven to be challenging for physicians. Currently, diagnosis is still based on the observation of a patient’s clinical features such as bradykinesia, resting tremor, or rigidity followed by the observation of a set of supportive criteria and the absence of a set of exclusion criteria outlined by the Movement Disorder Society Clinical Diagnostic Criteria for PD [1,2]. Furthermore, disease severity and progression are characterized using the Movement Disorder Society-Unified Parkinson’s Disease Rating Scale (MDS-UPDRS), which is also based on observation of clinical features associated with PD [3,4]. These types of assessments are subjective and prone to overlooking small changes in motor function that are representative of the disease, which poses challenges for PD screening [5]. This makes additional clinical tools that can be layered on to clinical evaluations by neurologists all the more critical [6]. An automated, point-of-care diagnostic platform that can be readily scaled in clinical and field settings could substantially advance screening capacity globally and detect PD earlier, leading to improved patient outcomes.

### 1.2. Advantages of Automation in Screening

While there is currently no reliable way to image Lewy bodies (the key pathological finding in PD patients), rapid advances in the precision and sophistication of technologies involved in the diagnosis of PD, from DaTscans to RT-QUIC to skin biopsies, have allowed for a more nuanced understanding of the disease and significant increases in the confidence of PD diagnosis [7,8,9]. However, there still remains a clear lack of readily accessible and sensitive PD screening methods that can be carried out prior to full-scale diagnostic evaluations by neurologists.

In recent years, smartphone and tablet technology has allowed for the potential development of high-throughput screening tools for PD. Automation provides several distinct advantages when it comes to disease screening and diagnosis, including low cost of use and enhanced speed and accessibility [10]. An automated screening tool can provide baseline information for neurologists and identify potential PD patients before symptoms bring them to a neurologist. This is particularly useful because there is a growing shortage of neurologists worldwide [11,12].

### 1.3. Handwriting Analysis for Parkinson’s Disease

Handwriting assessments for PD screening, in the form of serial signatures from pen-and-paper tests, were initially intended to detect micrographia, defined as abnormally small handwriting. Since micrographia and other kinematic variations in handwriting can be measured long before the onset of other PD symptoms, handwriting analysis has the potential to be an effective and scalable PD screening tool prior to formal clinical evaluation [13,14,15,16]. The earliest complex drawing tasks thereafter involved patients drawing a simple, circular clock (with the numbers one to twelve along the circumference) to screen for general neurological disorders [17]. With the development of computational tools and analytical methods in recent decades, handwriting analysis has become digitized to utilize kinematic data extracted from tablets during the writing process. This allows clinicians and researchers to quantitatively detect micrographia, tremors, and a variety of other PD symptoms. An analogous tool for Alzheimer’s disease screening, the DCTclock test^TM^, has demonstrated the feasibility of designing and implementing digital drawing tasks in clinical settings to assess areas of cognitive state. The DCTclock^TM^ test has demonstrated clinically significant associations for Alzheimer’s disease biomarkers as well as strong capabilities for discriminating between cognitively healthy individuals and patients with mild cognitive impairment or early Alzheimer’s dementia [18]. Passing clinical trials and receiving FDA clearance, the successful deployment of this tool speaks to the feasibility of a similar approach to PD screening as well as the readiness of modern clinical settings to incorporate these digitized methods. While qualitative analysis from a neurologist still serves as the standard for PD diagnosis, digitized handwriting and drawing tests have increasingly shown their potential in a clinical setting to be implemented as a cheap, easy-to-use, and accurate early-detection tool [19,20,21].

Beyond feasibility, digital handwriting analysis presents a number of clinical benefits for early screening and monitoring. Considering the familiarity of handwriting as well as the accessibility of a tablet-based tool, individuals can identify potential symptoms with greater ease and seek out a neurologist earlier. The collection of this data also serves to support a clinician’s potential diagnosis. Moreover, since motor symptoms of a PD patient are prone to fluctuate over time, readily available quantitative data on handwriting may aid clinicians in longitudinally monitoring patients and tracking the progression of disease. Personalized treatment strategies, novel drugs, and experimental approaches may also benefit from more extensive monitoring through digitized drawing tests due to their influence on a patient’s motor function [22,23].

### 1.4. Archimedean Spiral Drawings for Diagnosis of Parkinson’s Disease

Researchers have demonstrated the strong potential of digitized Archimedean spiral drawing tests in detecting motor dysfunctions associated with PD [24,25,26,27]. These tests focus on time-series kinematic and pressure measurements. Previous studies have designed batteries to detect features such as variability of spiral width, overall shape, spiral smoothness, pen velocity, and pen pressure. Advancements in digital spiral drawing, computerized feature analysis, drawing task design, and complex machine learning techniques established greater diagnostic ability, achieving impressive classification accuracies ranging from 79.78% to 97.52% [28,29,30,31,32,33,34,35] and area under the receiver operating characteristic curve (AUC) values ranging from 0.82 to 0.992 [33,36,37,38]. Research has continued to refine, augment, and standardize Archimedean spiral drawing assessments, exploring novel informative features for PD detection [39,40,41,42]. Our study builds on past studies involving the Archimedean spiral by combining spiral-specific features with traditional kinematic and pressure features to improve classification performance with simple and highly interpretable models.

### 1.5. Our Study

While existing studies have conducted the automation of spiral drawing analysis in PD patients, very rarely has the use of the Archimedean spiral been rationalized. As a result, many works neglect the unique features of the Archimedean spiral that make it powerful for PD diagnosis. We use these mathematical relationships inherent to the Archimedean spiral as bases of novel features, and we focus on important drawing metrics (such as pressure) to improve existing features in literature. These new features we propose are studied in addition to others described in the literature to create a comprehensive list of diagnostic features in PD spiral drawings. We then construct interpretable models with high diagnostic accuracy using these features. From this work and from existing investigations, we also provide guidelines and recommendations for future studies attempting to diagnose PD and other motor-coordination pathologies using digitized drawings so that this promising technique can eventually become a robust screening tool in the clinical setting.

## 2. Methods

### 2.1. The ParkinsonHW Dataset

**Figure 1:**
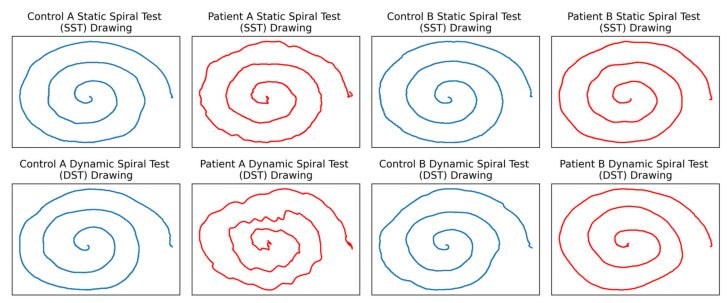
Sample of ParkinsonHW drawings. The top row of drawings is from the SST, and the bottom row is from the DST. Patients are colored in red, and controls are colored in blue. Unlike Patient A, Patient B would be difficult to diagnose visually due to the relatively normal drawing.

The ParkinsonHW dataset [43] contains kinematics, pressure, and pen angle-related data on Archimedean spiral (with three revolutions about the center) drawings performed on Wacom Cintiq 12WX graphics tablets with digital pens for 62 PD patients and 15 healthy controls. Three different types of the test were administered: the Static Spiral Test (SST; subjects trace a given spiral pattern), the Dynamic Spiral Test (DST; subjects trace a given blinking spiral pattern), and the Circular Motion Test (CMT; subjects draw circles around a red point) [43]. However, the majority of patients only performed SST and DST, with some only performing one of the tests. All healthy controls drew 2 spirals (SST and DST spirals) while 57 of the 62 patients drew DST spirals and 61 of the 62 patients drew SST spirals. Overall, 57 patients had two spiral drawings, 4 patients only had one spiral drawing, and 1 patient only performed the CMT (no spiral drawings). During each drawing, the x and y coordinates of the pen tip, the pen azimuth and altitude, the pressure exerted on the surface of the tablet by the pen tip, and the timestamp associated with each discrete data point are recorded. Figure 1 shows sample control and patient SST and DST drawings. Note that while some patients exhibit distinct motor dysfunctions (ex. Patient A), others draw almost just as well as the controls (ex. Patient B), increasing the difficulty of classification and motivating the use of digital data collection and modern machine learning techniques.

In evaluating the efficacy of the current model of tablet-based spiral drawings in differentiating between PD patients and healthy controls, we focused our analysis efforts on the SST and DST drawings, in which patients were given a fixed spiral pattern to trace.

### 2.2. Data Preprocessing

We first extracted the kinematic (velocity, acceleration, jerk) and curvature data from the raw data. Starting with the x and y coordinates, we performed 4th-degree univariate spline interpolation to smooth the signal and filter the noise present in the raw signals (Supplementary Section S1). We then computed derivatives of the spline-interpolated functions to get the desired kinematic data. Curvature of the subject’s drawing was also calculated.

Additionally, we calculated polar metrics of the spiral drawings. For each individual data point, we calculated the corresponding radius as the point’s distance from the center of the spiral and computed the point’s corresponding angle (referred to as theta in the rest of the paper) from the *x*-axis. Examples of polar features and Cartesian pre-processed features are included in Supplementary Materials Section S2.

We also broke down the pressure signal into three separate components: the rising edge, main signal, and falling edge (Supplementary Material Section S3).

### 2.3. Feature Engineering

To extract meaningful information, the kinematic, pressure, and angle-related data from the ParkinsonHW dataset were further processed into higher-order features. We included basic statistical measures for kinematic and pressure data signals, as well as interesting previously proposed features (normal velocity variability, entropy, skewness and kurtosis of raw data signals, rate of inversions in raw data signals, overall time duration, and time duration and numerical range of components of the pressure signal). We also constructed novel curvature, regression, and Fourier-transform-based features leveraging the unique mathematical properties of the Archimedean spiral. The complete list of features calculated is summarized in Supplemental Table S1. The details of each feature are described in Supplementary Material Section S4.

### 2.4. Feature Importance

For both static and dynamic features, the Mann–Whitney U Test was used to compare the distributions of each feature between patient and control groups. The *p*-value was used to assess the discriminative capacity of features. In addition, the AUC of each feature was calculated using the basic definition of AUC, which is the probability that a randomly selected patient has a higher value of the feature in comparison to a randomly selected control.

After extracting features from both the static and dynamic datasets, we selected the most informative features for further analysis using strict significance cutoffs at a maximum *p*-value of 1 × 10^−5^ or a minimum AUC of 0.9 to account for the testing of multiple features.

### 2.5. Subject Classification

Facing a heavy class imbalance, we chose to use the random forest classifier: a widely used machine learning technique employing an ensemble of decision trees. During classification, each decision tree returns a class prediction, and the random forest returns the class receiving the most such “votes”. With each of the individual decision trees trained on a different data subset (bootstrapping) and on a different feature subset (feature randomness), the random forest classifier prevents the overall model from relying too heavily on a set of values or features. Therefore, this classifier is especially applicable to our task because it is relatively robust against outliers and noise, can help reduce overfitting, and automatically weighs and selects useful features via decision trees [44].

Additionally, to avoid overfitting, we used a small training set, used a small number of predictor variables, and performed cross-validation. In particular, we aimed to improve generalization and reduce model complexity by discovering sparse feature subsets which are highly informative, rather than simultaneously applying a large number of features to our relatively small dataset. To this aim, we trained random forest classifiers containing at most 2 features, identified by the individual AUCs and Mann–Whitney U tests. We used 100 trees and the default values in sklearn version 0.24.2 for the other parameters in our classifier. Changing the parameters did not appreciably change the results. The strongest-performing feature sets are provided in the Section 3. For each set of features and for both the static and dynamic tests, the classifier was run 50 times for cross-validation, each time with a different random state and train-test sets, using a 50:50 train-test-split. Moreover, our models used features from either the SST or the DST (not both), so it is impossible for a subject to be represented in both the train and test set. The mean AUC, accuracy, precision, recall, and F-score of the classifier over the 50 runs is then calculated as the evaluation criteria for the effectiveness of that set of features.

All of the analyses were conducted using Python 3.7.1. Pandas, NumPy, SciPy and SciKit-Learn libraries were used for data pre-processing, feature calculations, and model construction. The use of specific libraries is discussed in their corresponding sections in the Supplementary Materials.

## 3. Results

### 3.1. Feature Selection

We extracted a total of 79 features, each of which were calculated for both the static and dynamic spirals. Out of the 79 features per sample, a total of 7 static features and 15 dynamic features satisfied the *p*-value cutoff of 1 × 10^−5^ described in the Methods. Similarly, a total of 5 static features and 11 dynamic features satisfied the 0.9 AUC cutoff. Table 1 gives a summary of these “significant” features.

### 3.2. Feature Visualizations

**Figure 2:**
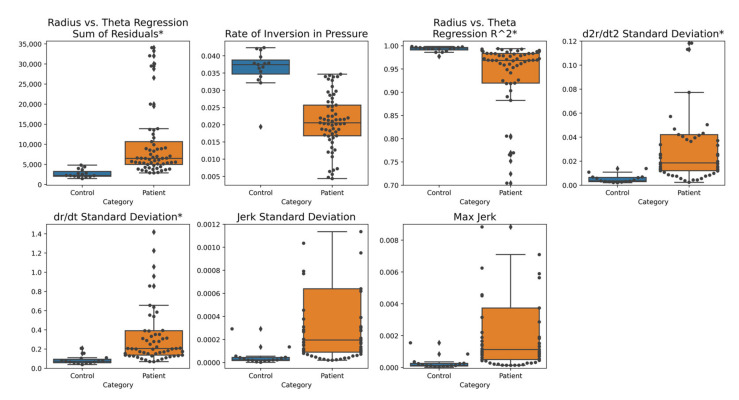
Static spiral test most informative features based on Mann–Whitney U test overlaid box and swarm plots. The distribution of each feature is visualized for patients and controls separately. Controls are on the left of each sub-figure and have a blue box plot. Patients are on the right of each sub-figure and have an orange box plot. Within this figure, we excluded sets of outliers in the “Radius vs. Theta Regression R^2^”, “$\frac{d^{2}r}{dt^{2}}$ Standard Deviation”, “$\frac{dr}{dt}$ Standard Deviation”, “Jerk Standard Deviation”, and “Max Jerk” feature plots to improve visibility of the separation between patients and controls. Newly proposed features are denoted with an asterisk (*).

**Figure 3:**
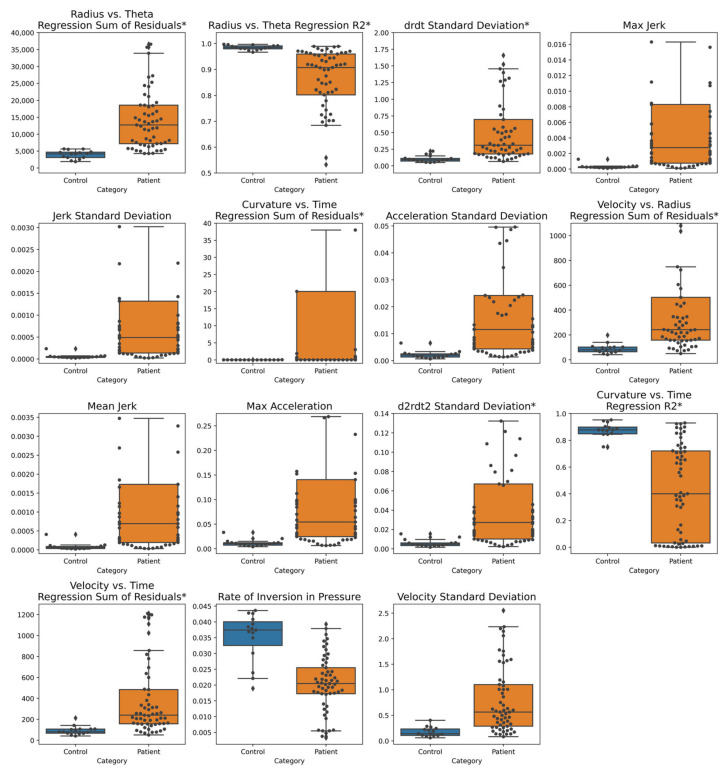
Dynamic spiral test most informative features based on Mann–Whitney U test overlaid box and swarm plots. The distribution of each feature is visualized for patients and controls separately. Controls are on the left of each sub-figure and have a blue box plot. Patients are on the right of each sub-figure and have an orange box plot. Within this figure, we excluded sets of outliers in every feature plot except the “Curvature vs. Time Regression R2”, “Rate of Inversion in Pressure”, and “Velocity Standard Deviation” feature plots to improve visibility of the separation between patients and controls. Newly proposed features are denoted with an asterisk (*).

We created overlaid box plots and swarm plots to visualize the discriminative powers of our most informative features, as listed in Table 1, for static spiral tests (Figure 2) and dynamic spiral tests (Figure 3). See Supplementary Material Section S5 (Static Features Visualization) and Supplementary Material Section S6 (Dynamic Features Visualization) for visualizations of other novel features that were not designated as the most informative.

Across the most informative features, we see clear visual separation between the patient and control data distributions, as the medians substantially differ, and interquartile ranges do not overlap. Additionally, feature value distributions for patient populations generally exhibit much greater variabilities than those of control populations.

**Figure 4:**
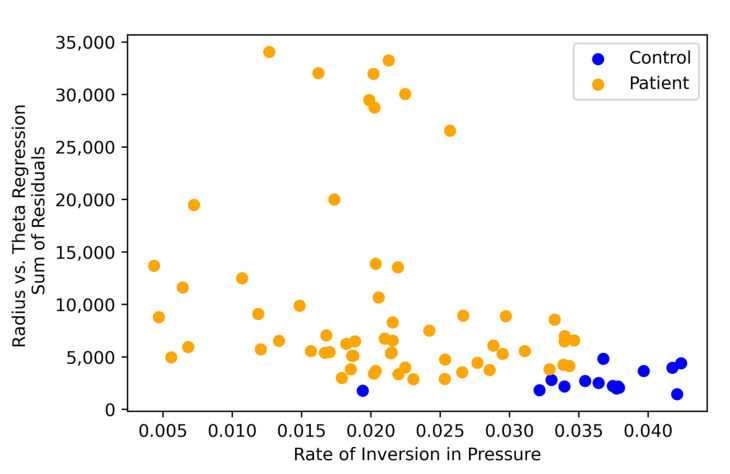
Plot of the two most informative features for the static drawing test. Each point represents a subject. The rate of inversion in pressure is plotted against the radius vs. theta regression sum of residuals for each subject. Orange points are patients and blue points are controls.

In addition to examining individual features, we consider patient and control distributions across pairs of features to visualize the separability identified by our top-performing models. In Figure 4, we display the top two features from the static drawings as identified by the *p*-value and individual AUC cutoffs (rate of inversion in pressure and radius vs. theta regression sum of residuals). We see nearly linear separability between classes in these two dimensions.

### 3.3. Classification Results

The selected feature sets and random forest classification AUCs are summarized in Table 2. We included the top performers for the static and dynamic tests with only two features. We also show highly informative individual features to understand classification accuracy with the simplest of models. The highest performing model for the static spirals had an AUC of 0.999, accuracy of 0.976, precision of 0.986, F-score of 0.985, and recall of 0.984. The highest performing model for the dynamic spirals had an AUC of 0.996, accuracy of 0.964, precision of 0.987, F-score of 0.977, and recall of 0.969.

## 4. Discussion

### 4.1. Discussion of Classification Results

#### 4.1.1. Model Accuracy

Instead of evaluating many features that are mildly associated with PD, we focused on engineering specific features that are strongly indicative of PD on which to base our model construction. Using only two features of different categories, we were able to train remarkably accurate models, producing AUCs of 0.999 and 0.996 for the static and dynamic tests respectively and accuracies of 0.976 and 0.964 for the static and dynamic tests respectively. These performance metrics are greater than or equivalent to the maximum AUC of 0.992 and accuracy of 0.975 currently reported in the literature [32,33]. Our model also uses relatively small training data sizes to ensure the robustness of the features. Most importantly, in comparison to past studies that trained models on larger feature sets, created complex models, and used black box like approaches to patient classification, our study, which uses simple models, confers more interpretability, allowing researchers to pinpoint the specific diagnostically powerful features. This characteristic will help clinicians better understand and utilize drawing-based screening tools.

#### 4.1.2. Important Features

Features that achieved particularly high classification AUCs by themselves and in conjunction with other features include Rate of Inversion in Pressure, Radius vs. Theta Regression R^2^, Radius vs. Theta Regression Sum of Residuals, $\frac{d^{2}r}{dt^{2}}$ Standard Deviation, Velocity vs. Radius Regression Sum of Residuals, and Curvature vs. Time Regression Sum of Residuals (Table 2).

Examining individual features, the Rate of Inversion in Pressure was observed to be significantly lower among patients than controls. This result, although counterintuitive—since we expect tremors and large degrees of variation in pressure among the patient population—can be attributed to how pressure changes during tremors are captured by the pencil. Since the sampling frequency of the pen is greater than the tremor frequency [45,46], the pressure signal during a tremor will be recorded as a series of increasing pressure values followed by a decreasing series (or vice versa), amounting to only a single inversion in pressure at the local extremum. In contrast, a sample of pressure values spanning the same amount of time taken from a control drawing will likely produce many pressure values that are very close in magnitude but fluctuate randomly rather than systematically increase and decrease, so several inversions in pressure are recorded. In other words, actual patient tremors are distinctly captured, while random variations in the relatively smooth control pressure signals contribute to a large number of insignificant, fine inversions.

The radius vs. theta regression R^2^ feature was observed to be significantly lower among patients than controls. Since the radius of an Archimedean spiral varies directly with its angle about the origin, the R^2^ value of the linear regression between the two features of a drawing indicates the degree to which the drawing deviates from a perfect spiral form. The tremors of patients and other abnormalities in handwriting resulted in drawings that varied more from the spiral template, producing a lower Radius vs. Theta Regression R^2^ value on average. For the same reason, the Radius vs. Theta Regression Sum of Residuals, was observed to have significantly higher values among patients than controls. Further explanations of the other informative features are provided in Supplementary Materials Section S7 (Explanation of Further Informative Features).

In practice, conventional qualitative tests rely upon baseline heuristics which may capture clearly-presenting symptoms characteristic of mid-stage PD patients. It is unlikely that the same broad heuristics would be sufficient to capture more subtle motor symptoms associated with early-stage neuromotor conditions. To reduce the incidence of undetected symptoms and increase early diagnoses, having a test with greater sensitivity to these “hidden” symptoms is necessary.

**Figure 5:**
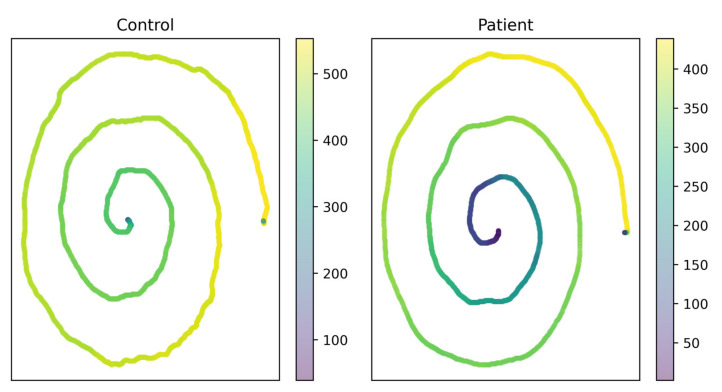
Spiral drawings colored by pressure. Visual classification is difficult, but our model classifies these subjects correctly. The difference in pressure profiles is shown clearly in the patient (**right**) and control (**left**). Darker areas are regions of low pressure and lighter areas are regions of high pressure.

It seems that our data analysis pipeline could be well-suited for this role. Notably, our models seem to extract enough useful information from higher-order features to correctly classify particularly tricky test cases. Figure 5 depicts an example of a control spiral which is visibly more shaky than a patient spiral; visual inspection would indicate that the control spiral on the left is more likely to have been drawn by a PD patient. Despite this, our trained models were consistently able to use indicative factors gleaned from higher-order features to correctly classify both spirals.

### 4.2. Future Diagnostic Recommendations

#### 4.2.1. Drawing Shape Recommendations

The choice of drawing shapes is one of the most important aspects of drawing test design. We have reasoned that shapes with distinctive mathematical relationships fit well with quantitative analyses of digitized drawings. Deviations from these mathematical relationships inherent to the drawing figure can be used to distinguish between patient and control, or even assess the stage of disease progression. The Archimedean spiral used in this study is an example of such a mathematically interesting shape, featuring unique curvature characteristics and distinctive relationships between radius and theta about the origin (see more detail in the Section 2). Other mathematically interesting shapes include those with nonconstant curvatures such as lemniscates and spirographs—the variations in their curvatures could be diagnostically important.

Unorthodox shapes are also suitable for use, as they “normalize” for the level of education and drawing experience among the test subjects. Examples are the Poppelreuter-type figures or other figures that subjects have not encountered before. This class of shapes becomes especially beneficial for more cognitive and memory-based diseases such as Alzheimer’s Disease, but they can also be beneficial for revealing PD-associated motor function as well.

#### 4.2.2. Template for Drawing

The structure and guidance provided to the patient during the drawing task is a tricky matter of balance. Options include tracing over shapes, giving general guidelines such as flashing lines or moving dots, and simply providing an empty drawing pad for freehand drawings. While freehand clock-drawing, when used in the mini-cog test and in an automated test performed by MIT scientists, have been found to be indicative of PD [47,48], Drotar et al. have shown that the large degree of variation in freehand spiral drawings significantly confounds and complicates the analysis [28]. Our preliminary feature-extraction efforts based on the PaHaW dataset collected by Drotar et al. have also confirmed the difficulty in analyzing freehand spirals due to the high variability in these spiral drawings. This variability makes it more challenging to identify disease-associated features.

On the other hand, simple tracings have been proven to be useful in detecting PD [49]. The standardization of tracings through drawing templates (including for spirals and other shapes) dramatically improves drawing uniformity and creates clearer distinctions between patient and control. Finding the middle ground, the presence of some guidance without pure tracing may create enough inter-patient variability while still allowing for some uniformity, which will allow current analytic methods to detect Parkinsonian features.

#### 4.2.3. Adaptive Framework

The rigidity of drawing tasks with only a single standardized shape can be improved upon [29]. It largely fails to account for different stages of PD [50], different natural drawing abilities [41], and different circumstances, from levels of cognitive ability [51] and work experience to technological literacy, especially in older adults who are more likely to be tested for PD. As people with PD in its earlier stages will have vastly different clinical needs than those with PD in later stages, grouping patients in both of these situations in the same category will not be beneficial to patients who want to better understand their disease and for doctors interpreting the results [52,53]. These concerns warrant the development of drawing tests with varying difficulties. For future studies, an excellent structure for such drawing tests is the adaptive testing framework, in which patients are given drawing tasks of varying difficulty based on their performance on a previous drawing task. For instance, one would have the patient draw a shape and if they draw it well, have them draw a more complex shape. If they draw it poorly, have them draw a simpler shape. Such adaptive testing potentially allows for clearer differentiation between different stages of PD. 

#### 4.2.4. Task Variation

Beyond utilizing adaptive testing frameworks that consider patient abilities for more nuanced assessments, drawing tasks can leverage repetitive evaluation (measurement over multiple trials) on the same shape to gather informative data on drawing variability and also the learning rate of patients. Drawing tasks can also incorporate variations in the form of testing conditions, such as requiring the patient to draw a shape in multiple trials with different objectives or obstacles. The conditions may range from emphasizing accuracy or speed to displaying intermittently flashing template lines for tracing. These deviations from a patient’s previously completed drawing tasks will likely allow for easier differentiation between PD patient and control data [54,55].

#### 4.2.5. Data Sharing

There have been many studies that have collected digitized drawing/handwriting from PD patients. However, datasets used in the majority of studies are not publicly available and have very small sample sizes (often less than 50 total subjects). Models trained on such small datasets will likely be biased with possibly limited generalizability [56]. Additionally, non-publicly available datasets hinder the possibility of external validation on similar handwriting or drawing tasks [57]. To address these issues, combining datasets from heterogeneous populations can help reduce imbalances in race, gender, age, etc. that will impact model classification accuracies on general populations. Moreover, making public datasets with credentialed access and de-identified data information is important so that larger groups of researchers can come together to verify that automated drawing analysis is a valid and accurate PD screening method. These datasets could even be made available on the Michael J. Fox Foundation datasets page [58].

#### 4.2.6. Limitations

While we have achieved extremely high AUCs with our feature engineering and model construction methods on the ParkinsonHW dataset, it is important to validate the generalizability of our approach, especially given the class imbalance in the ParkinsonHW dataset (low number of control drawings) and the relatively low overall sample size. Moreover, there may be possibilities for early detection of PD using this automated method, but there are currently no datasets or studies that can robustly prove early detection capability. The lack of MDS-UPDRS scores or similar disease progression scores is another limitation. Future studies should aim to collect MDS-UPDRS from many patients, so that we can adequately test automated detection of disease severity.

In addition, considering the significant overlap of PD symptoms with those of other neurodegenerative diseases and atypical Parkinsonian disorders, differentiation requires that assessments leverage subtle quantitative features to construct powerful tools for clinicians [59]. This necessity has remained the subject of continuous study with numerous proposed solutions, and the digitization of screening assessments has only further complicated the discussion. Tremor variation over time during resting tasks, modified shape-drawing, and supplementary imaging alongside alternative tests are among the many solutions pursued in previous literature in order to differentiate PD motor symptoms from those of conditions such as essential tremor, multiple system atrophy, and Lewy body dementia (LBD) [60,61,62]. To separate PD from progressive supranuclear palsy, a frequently-encountered cause of atypical parkinsonism, studies have presented suggestions ranging from the identification of hypokinesia without decrement in handwriting tasks to the extraction of characteristic features through gait analysis [63,64]. In addressing dementias such as Alzheimer’s disease and LBD, their visuospatial symptoms, which influence patient handwriting, have been the subject of ongoing research due to their striking similarities to symptoms of PD [65,66]. Distinguishing between PD and similar conditions continues to present a major obstacle to the development of diagnostic tools, requiring further research. Certain features we have extracted will be more associated with a general abnormal state such as radius vs. theta regression R^2^ as it captures overall drawing ability. Other, more nuanced features, such as rate of inversion in pressure and curvature regression may be better suited to differentiate between PD and similar conditions.

## 5. Conclusions

By identifying intuitive and highly predictive features in spiral drawings from PD patients, we have been able to differentiate PD patients from controls with a very high AUC. While improvements must be made to the drawing task and study design to rigorously determine the accuracy of this method for PD diagnosis, this study demonstrates the potential of this method to enable wide-spread, point-of-care PD screening. In the face of increasing prevalence of PD and decreasing numbers of neurologists worldwide, tools for screening for the disease will be imperative for managing PD, particularly in under-resourced areas. By developing and validating low-cost, automated handwriting-based screening technologies for PD, community health care workers and primary care clinics could rapidly identify patients at-risk for PD, reducing the burden of PD globally.

## Figures and Tables

**Table 1:** Most informative features. For both static and dynamic drawings and using AUC and *p*-value as metrics, we identified the most informative features for patient classification. The features marked with asterisks (*) are our newly proposed features. r = radius, t = time.

	*p*-Value Cutoff	AUC Cutoff
Static Drawing	Radius vs. Theta Regression Sum of Residuals * Rate of Inversion in Pressure Radius vs. Theta Regression R^2^ * $\frac{d^{2}r}{dt^{2}}$ Standard Deviation * $\frac{dr}{dt}$ Standard Deviation * Jerk Standard Deviation Max Jerk	Radius vs. Theta Regression Sum of Residuals * Rate of Inversion in Pressure Radius vs. Theta Regression R^2^ * $\frac{d^{2}r}{dt^{2}}$ Standard Deviation * $\frac{dr}{dt}$ Standard Deviation *
Dynamic Drawing	Radius vs. Theta Regression Sum of Residuals * Radius vs. Theta Regression R^2^ * $\frac{dr}{dt}$ Standard Deviation * Max Jerk Jerk Standard Deviation Curvature vs. Time Regression Sum of Residuals * Acceleration Standard Deviation Velocity vs. Radius Regression Sum of Residuals * Mean Jerk Max Acceleration $\frac{d^{2}r}{dt^{2}}$ Standard Deviation * Curvature vs. Time Regression R^2^ * Velocity vs. Time Regression Sum of Residuals * Rate of Inversion in Pressure Velocity Standard Deviation	Radius vs. Theta Regression Sum of Residuals * Radius vs. Theta Regression R^2^ * $\frac{dr}{dt}$ Standard Deviation * Max Jerk Jerk Standard Deviation Curvature vs. Time Regression Sum of Residuals Acceleration Standard Deviation Velocity vs. Radius Regression Sum of Residuals * Mean Jerk Max Acceleration $\frac{d^{2}r}{dt^{2}}$ Standard Deviation *

**Table 2:** Random forest classification performance using different sets of features. The AUC for both static and dynamic tests are displayed for informative feature sets and individual features. The highest AUCs for the static and dynamic tests are bolded.

Features	Static AUC	Dynamic AUC
Rate of Inversion in Pressure, Radius vs. Theta Regression Sum of Residuals	**0.999**	0.975
Curvature vs. Time Regression Sum of Residuals, Radius vs. Theta Regression Sum of Residuals	0.894	**0.996**
Rate of Inversion in Pressure	0.934	0.779
Radius vs. Theta Regression R^2^	0.910	0.906
Radius vs. Theta Regression Sum of Residuals	0.887	0.956
Curvature vs. Time Regression Sum of Residuals	0.666	0.911
Velocity vs. Radius Regression Sum of Residuals	0.675	0.843

## Data Availability

The open-access data used for this study are available here: https://archive.ics.uci.edu/ml/datasets/Parkinson+Disease+Spiral+Drawings+Using+Digitized+Graphics+Tablet, accessed on 15 November 2020. Code can be provided upon request.

## Supplementary Materials

The following are available online at https://www.mdpi.com/article/10.3390/brainsci11101297/s1. Figure S1: Sample pre-processed data signals. Figure S2: Sample pressure signal breakdown into rising edge, main signal, and falling edge. Figure S3: Template Archimedean spiral and its corresponding curvature. Figure S4: Novel curvature-based features overlaid box and swarm plots for static spiral tests. Figure S5: Novel Fourier transform-based features overlaid box and swarm plots for static spiral tests. Figure S6: Novel linear regression-based features overlaid box and swarm plots for static spiral tests. Figure S7: Overlaid swarm and box plots for the novel features based on the inversely proportional relationship between velocity and curvature for static spiral tests. Figure S8: Overlaid swarm and box plots for the novel features based on the rates of change of radius and theta for static spiral tests. Figure S9: Overlaid swarm and box plots for the novel features based on the rates of change of radius with respect to theta for static spiral tests. Figure S10: Novel curvature-based features overlaid box and swarm plots for dynamic spiral tests. Figure S11: Novel Fourier transform-based features overlaid box and swarm plots for dynamic spiral tests. Figure S12: Overlaid box and swarm plots for novel linear regression-based features for dynamic spiral tests. Figure S13: Overlaid swarm and box plots for the novel features based on the inversely proportional relationship between velocity and curvature for dynamic spiral tests. Figure S14: Overlaid swarm and box plots for the novel features based on the rates of change of radius and theta for dynamic spiral tests. Figure S15: Overlaid swarm and box plots for the novel features based on the rates of change of radius with respect to theta for dynamic spiral tests. Table S1: Complete list of features calculated.
