# Supplementary material for: Screening of Parkinson’s Disease Using Geometric Features Extracted from Spiral Drawings
Source: Brain Sci. 2021 Sep 29;11(10):1297. doi: 10.3390/brainsci11101297 (PMC8533717; doi:10.3390/brainsci11101297)
Supplement: Supplementary file 1 [file brainsci-11-01297-s001.zip › supp_material_revised.pdf]

Supplementary Material

1. Spline Fitting

After testing the processed data signals, we used different degrees of smoothing for different types of signals. The smoothing factor, which dictates the number of knots for the spline fit, is 10,000 for velocity, acceleration, and jerk and 100,000 for curvature. As a result of smoothing, the data signals of all drawings, which originally differed from each other in length, were resampled to contain 1,000 points (since the drawing with the least number of original data points had about 1,000 data points). We then removed the first 50 and the last 50 data points from the velocity, acceleration, and jerk signals, and removed the first 50 and the last 100 data points from the curvature signals to ignore the irregularities at the beginning and end of the data collection process. For the radius and θ calculations, we resampled to 1,000 data points, and filtered through a smoothing factor of 1,000. All of the 4th degree spline fits are created with the SciPy v1.6.3 UnivariateSpline function.

2. Pre-Processed Data Visualization

Note that the raw data signals that are directly used as features (i.e. pressure and penaltitude) were not smoothed and do not have the beginning and end points removed.

Supplemental Figure 1 provides visualization for the sample data for each of the features (kinematic, pressure and altitude) for both SST and DST drawings of a single Parkinson's patient.

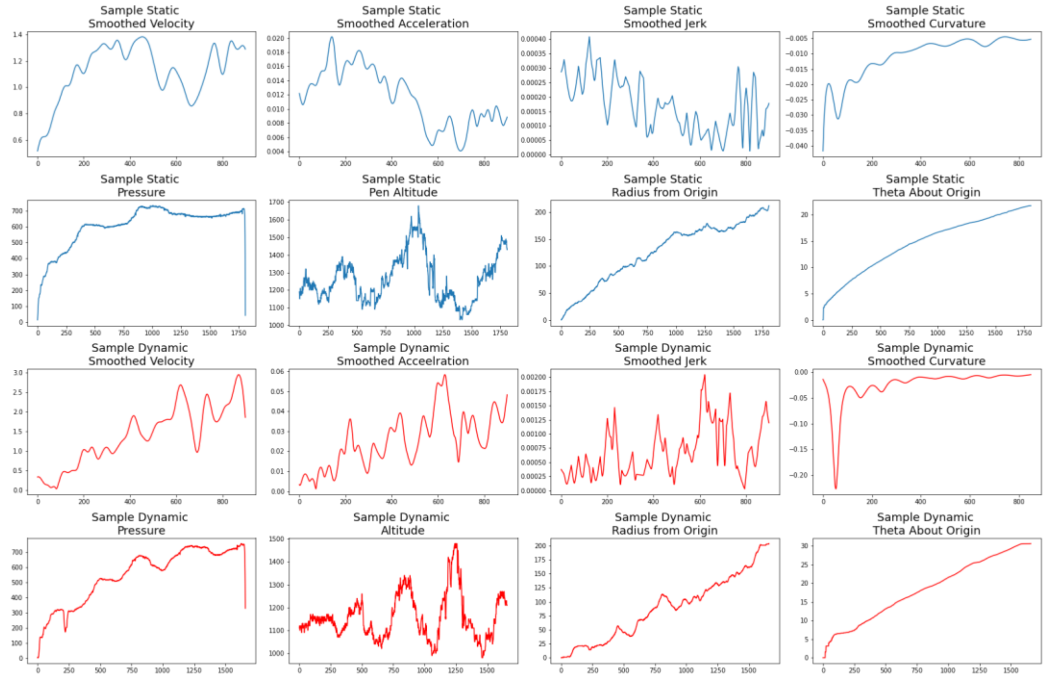

Figure S1. Sample Pre-processed Data Signals. The top 8 subfigures in blue show results for the static test. The bottom 8 subfigures in red show results for the dynamic test. Each set of subfigures displayed the smoothed velocity, acceleration, jerk, curvature and the original pressure, pen altitude, radius from the origin, and theta about the origin.

3. Pressure Rising Edge, Main Signal, and Falling Edge

This distinct three-component pattern exists across all pressure signals from Archimedean spiral drawings, including those from other datasets. In addition to providing insights about how the subjects start and end the drawing through the rising and falling edges, such a breakdown enhances analysis of the main signals, eliminating the influence of heavy low-end outliers at the edges. The component breakdown is accomplished by simply pinpointing where consecutive points stop increasing (for rising edge) and where consecutive points start decreasing (for falling edge). More specifically, these points were determined by using thresholds for the ratio between pressure values at different times to identify a significant increase or drop (1.01 for the detection of the end of the rising edge and 0.7 for the detection of the start of the falling edge). A significant drop in pressure corresponds to the start of the falling edge and the flattening in pressure from regions of pressure increase corresponds to the start of the main signal.

Supplemental Figure 2 demonstrates a sample breakdown, with the red part corresponding to the rising edge, the blue part to the main signal, and the green part to the falling edge.

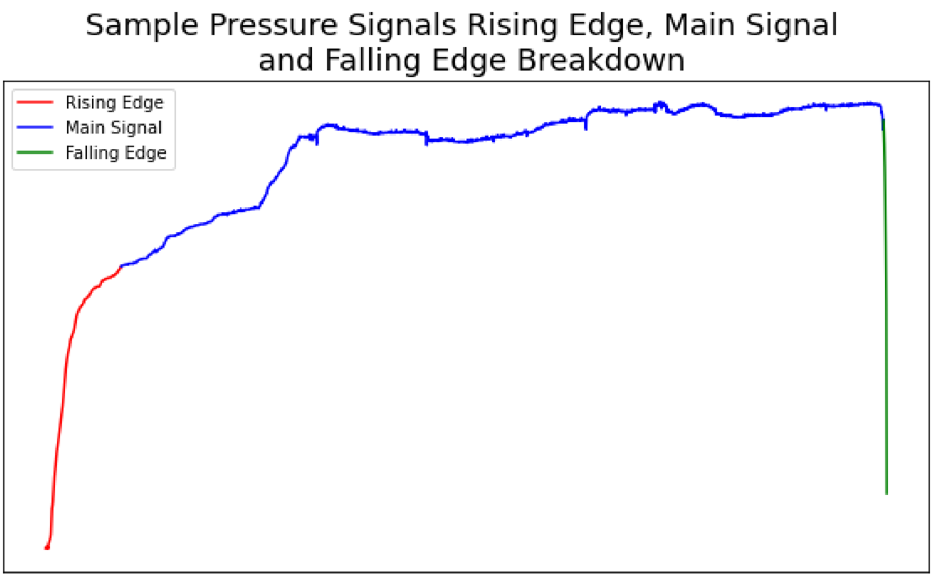

Figure S2. Sample Pressure Signal Breakdown into Rising Edge, Main Signal,& Falling Edge. The first red portion of the pressure signal is the rising edge, which is the pressure as the subject begins to draw. The main signal, colored blue, is the pressure while the subject is actively drawing the spiral. The final position in green is the falling edge, which is thepressure as the subject is picking up the pen.

4. Features

Novel Features

1. Curvature

We combined existing and novel methods to analyze a newly proposed feature - the curvature of the Archimedean spiral. Mathematically, curvature is the degree to which a curve deviates from being a straight line, and the curvature of a perfect Archimedean spiral drawn in the clockwise direction starts at a negative value and increases from beginning to end.

Supplemental Figure 3 illustrates the template ("perfect") curvature curve of an Archimedean spiral. In general, Parkinson's patients demonstrate abnormal motor control and exhibit jerkier motions when drawing, resulting in heavy deviations from the "template" spiral and curvature.

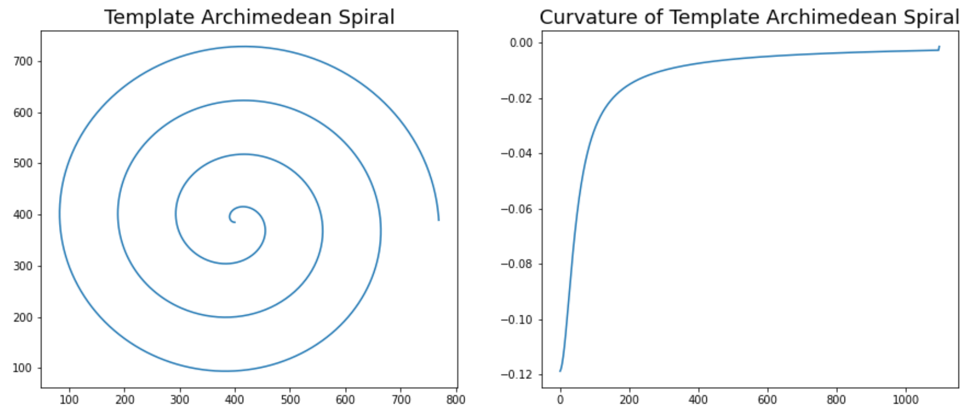

Figure S3. Template Archimedean Spiral & Its Corresponding Curvature.

We developed novel curvature-based features. For plane curves parameterized as $r(t) = x(t)\hat{\imath} + y(t)\hat{\jmath}$, the curvature is defined as follows.

$$\kappa = \frac{|x''y' - x'y''|}{((x')^2 + (y')^2)^{3/2}}$$

The polar form of the equations is then,

$$\kappa = \frac{|2((f'(\theta))^2| + ((f(\theta))^2 - f(\theta)f''(\theta))}{[((f'(\theta))^2 + ((f(\theta))^2]^{3/2}}$$

An Archimedean spiral can be defined in polar form as $r = f(\theta) = a + b\theta$, and therefore $f'(\theta) = b$ and $f''(\theta) = 0$. We substitute these expressions above in the polar form of the curvature equation. Then, since the dominant term in the numerator is on the order of $\theta^2$and the dominant term in the denominator is θ^3, we approximated κ to have a roughly inverse relationship to θ. We also make the approximation that $\theta(t)$ is linear in t. Altogether, we modeled curvature as the following inverse relationship: $\kappa = c + \frac{d}{t}$. We then use regression to solve for the constants c and d. From the constructed regression model, we extracted the R-squared value, the equation coefficients (c and d as modeled in $\kappa = c + \frac{d}{t}$), and the sums of squared residuals as features.

2. Fourier Transform (Relative Amounts of High & Low Frequency Content)

Fourier Transform is a signal analysis technique that involves decomposing a signal in the time domain into its underlying sinusoidal functions and phase shifts for analysis in the frequency domain. Applying this transformation to handwriting signals enables extraction of the relative amounts of high, low, and medium frequency content in the signal, which we defined by setting different frequency cutoffs expressed as a fraction of the Nyquist frequency (70Hz).

Specifically, we chose a fractional cutoff of 0.12 (8.4Hz) to separate the low and high ranges, and a cutoff range of 0.05 to 0.20 (3.5Hz to 14Hz) to define the middle range. This information can provide insight into the presence of tremors for Parkinson's patients compared to healthy controls. These spectral features were extracted from the pressure and altitude signals. Higher frequency content in pressure can be indicative of tremors in fine motor control, and high frequency variations in altitude can be indicative of abnormal variations in pen grip. Thus, we expect Parkinson's patients to produce higher values of relative high frequency content in their Fourier-transformed pressure and altitude signals than those of controls.

In our analysis, we used SciPy's (v1.6.3) butter function to create a 5th order Butterworth filter. We then applied the filter to our data using SciPy's filtfilt function with default parameters.

3. Linear Regression-Based Features

The following properties of both the Archimedean spiral itself and of drawing the Archimedean spiral motivate linear-regression-based modeling:

1. In polar coordinates (r, θ), an Archimedean spiral can be described by the equation $R(\theta) = a + b\theta$. The coefficient a controls the position of the center of the spiral, and the coefficient b controls the distance between the spiral loops.
2. When drawing curved trajectories, the velocity is shown to be proportional to the radius of curvature of the trajectory ($v = ar$, where v is the velocity and r is the radius) [1]. This relationship also reveals that velocity and time roughly demonstrate a directly proportional relationship, as radius is directly proportional to time in a drawing.
3. From a physiological standpoint, when a subject draws an Archimedean spiral from the inside out, pressure tends to increase with the radius of the spiral as the velocity also increases.

Utilizing these properties, we applied linear regression to four sets of data: radius vs. theta, velocity vs. radius, velocity vs. time, and pressure (main signal) vs. time. Four quantitative measures of linear regression fits are used as features: the regression coefficient of determination (R-squared values), y-intercept of the regression line, slope of the regression line, and the sum of squared residuals of the regression. The differences between patients and controls in the R-squared and sums of squared residuals metrics may reveal the patients' tendencies to draw with more randomness and less predictability. The regression models were built with the StatsModels v0.12.2 library's OLS function using default parameters

4. Inversely Proportional Relationship between Velocity & Curvature

As mentioned above, since radius and velocity are directly related, and since radius and curvature are inversely related, velocity and curvature are inversely related. Therefore, we constructed a regression model based on the relationship $v = a + \frac{b}{\kappa}$, where v is the velocity and κ is the curvature. From there, we use the R-squared value, the equation coefficients (a and b as modeled above), and the sums of squared residuals of the regression model as features. The models were built with Scipy's (v1.6.3) curve_fit function using default parameters.

5. Mean & Standard Deviation of the Rates of Change of Radius & Theta

Utilizing the polar properties of the Archimedean spiral, we analyzed the first and second order derivatives of radius as a function of time and theta as a function of time. The radius and theta signals were calculated and then smoothed as described in the data preprocessing section, with radius being the distance from the center of the spiral and theta being the angle from the horizontal polar axis. The derivatives were calculated from the spline fitted functions of these data signals and provide the rate of change at each data point in the smoothed and truncated drawing. The mean and standard deviations of the rate-of-change values provide insight into the stability of the subject's drawing. We would expect patients to demonstrate higher rates of change (greater mean values) and more variability (higher standard deviation) in the rates of change of their drawings.

6. Mean & Standard Deviation of the Rate of Change of Radius with Respect to Theta

Another important novel feature is the rate of change of radius with respect to theta ($\frac{dr}{d\theta}$). From the polar equation of the Archimedean spiral ($r = a + b\theta$), $\frac{dr}{d\theta}$ corresponds to the coefficient b in the equation, and a perfect Archimedean spiral should maintain a

constant $\frac{dr}{d\theta}$value. We can extract meaningful information about the particular drawing's deviation from a perfect spiral through the mean and standard deviation values of the subject's $\frac{dr}{d\theta}$ distribution at each point of the drawing. We expect the mean $\frac{dr}{d\theta}$ value for healthy controls to be closer to the expected value, and for the standard deviation to be lower.

Previously Described Features

1. Normal Velocity Variability (NVV)

Introduced in a 2017 study by Kotsavasiloglou et al. [2], the normal velocity variability (NVV) feature characterizes the variability of the subject's drawing velocity and was shown to be potent in identifying pathological movement patterns. The feature can be calculated with the following formula:

$$NVV = \frac{1}{T|MV|} \cdot \sum_{i=1}^{N-1} |v_{i+1} - v_i|, \text{ where } MV = \frac{1}{N} \cdot \sum_{i=1}^{N} v_i$$

(where T is the total duration, N is the number of data points in the sample, and v denotes the magnitude of the particular velocity data point).

2. Shannon Entropy

We adapted the use of Shannon entropy as a feature from Drotár et al.'s 2015 study [3]. As a numerical measure of the randomness or uncertainty of a signal, Shannon entropy can reveal hidden complexities related to the physiological system in the handwriting process. We calculated Shannon entropies for a variety of time-series signals in our dataset, namely the x-coordinate, y-coordinate, velocity, acceleration, jerk, pressure, and curvature signals. TheShannon entropy formula is calculated as follows:

$$H_s(X) = -\sum_{x \in X} p(x) \, log_2 p(x)$$

(where $p(x)$ is the probability density function computed using kernel density estimation with a Gaussian kernel). To calculate Shannon entropy, we used sklearn's (v0.24.2) KernelDensity function with a bandwidth of 10 and a Gaussian kernel. Other parameters were setto default values.

3. Skewness & Kurtosis

The use of skewness & kurtosis as features was first proposed in 2014 by Drotár et al. [4]. Skewness provides a measure of the lack of symmetry in a data distribution, while kurtosis offers a measure of whether the data distribution is "heavy-tailed" or "light-tailed" compared to a normal distribution. We performed skewness & kurtosis calculations on a variety of signals, from the X and Y coordinates, to velocity, acceleration, jerk, pressure, and curvature signals.

4. Rate of Inversions

The number and rate of inversions, or the number and rate of changes in directions, have been widely used in Parkinson's handwriting analysis literature [5–7]. These features are applicable to a range of signals and provide potential indication of loss of fine motor control. We elected to use the rate of inversions in our study in order to normalize the number of inversions collected from signals of drastically different lengths in the ParkinsonHW dataset, dividing the raw number of inversions by the time duration of the corresponding drawing. We applied this feature to velocity, acceleration, jerk, pressure, & curvature signals.

5. Duration & Range of Pressure Rising Edge & Falling Edge

In 2016, Drotár et al. proposed calculating pressure features separately for rising edge, main signal, and falling edge components of the pressure signal, specifically the duration (maximal timestamp - minimal timestamp) and range (maximal pressure value - minimal pressure value) of the rising and falling components [8].

5. Static Features Visualization

The following series of figures present the overlaid box plots and swarm plots for the newly proposed features that didn't meet the previously mentioned selection criteria for the "most informative features." In this set of figures, the plots marked with asterisks (*) have sets ofoutliers excluded to enhance plot visibility.

Curvature-Based Novel Features

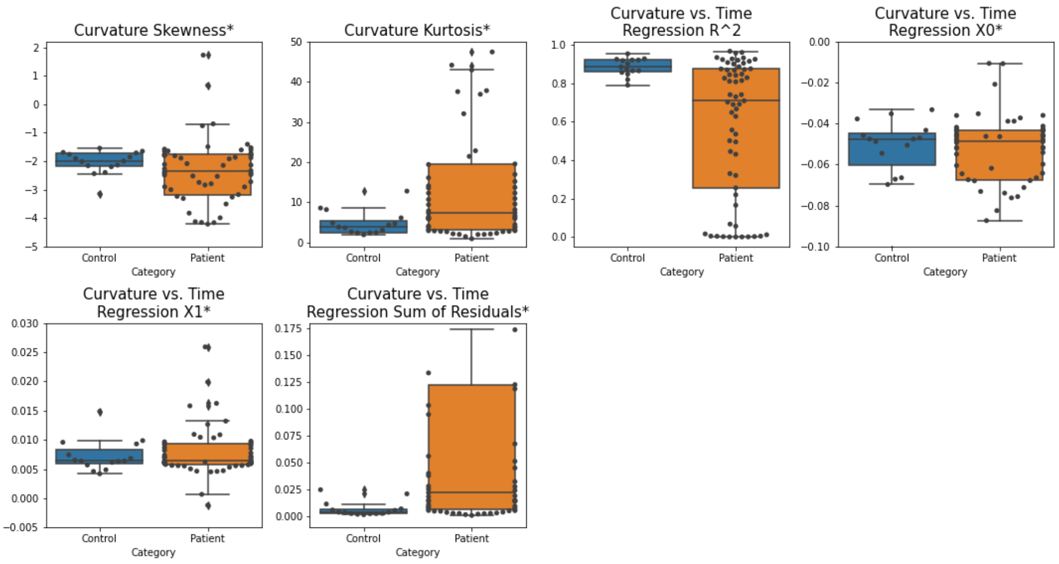

Figure S4. Novel curvature-based features overlaid box & swarm plots for static spiral tests. The distribution of each curvature feature is visualized for patients and controls separately. Controls are on the left of each sub figure and have a blue box plot. Patients are on the right of each sub figure and have an orange box plot.

Fourier Transform-Based Features

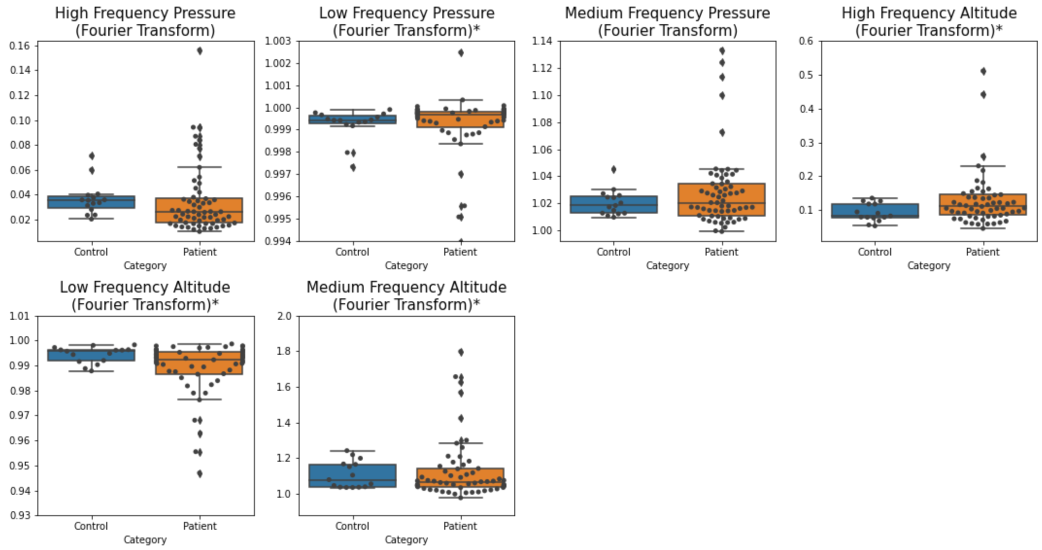

Figure S5. Novel Fourier transform-based features overlaid box & swarm plots for static spiral tests. The distribution of each Fourier transform-based feature is visualized for patients and controls separately. Controls are on the left of each sub figure and have a blue box plot. Patients are on the right of each sub figure and have an orange box plot.

Linear Regression Based Features

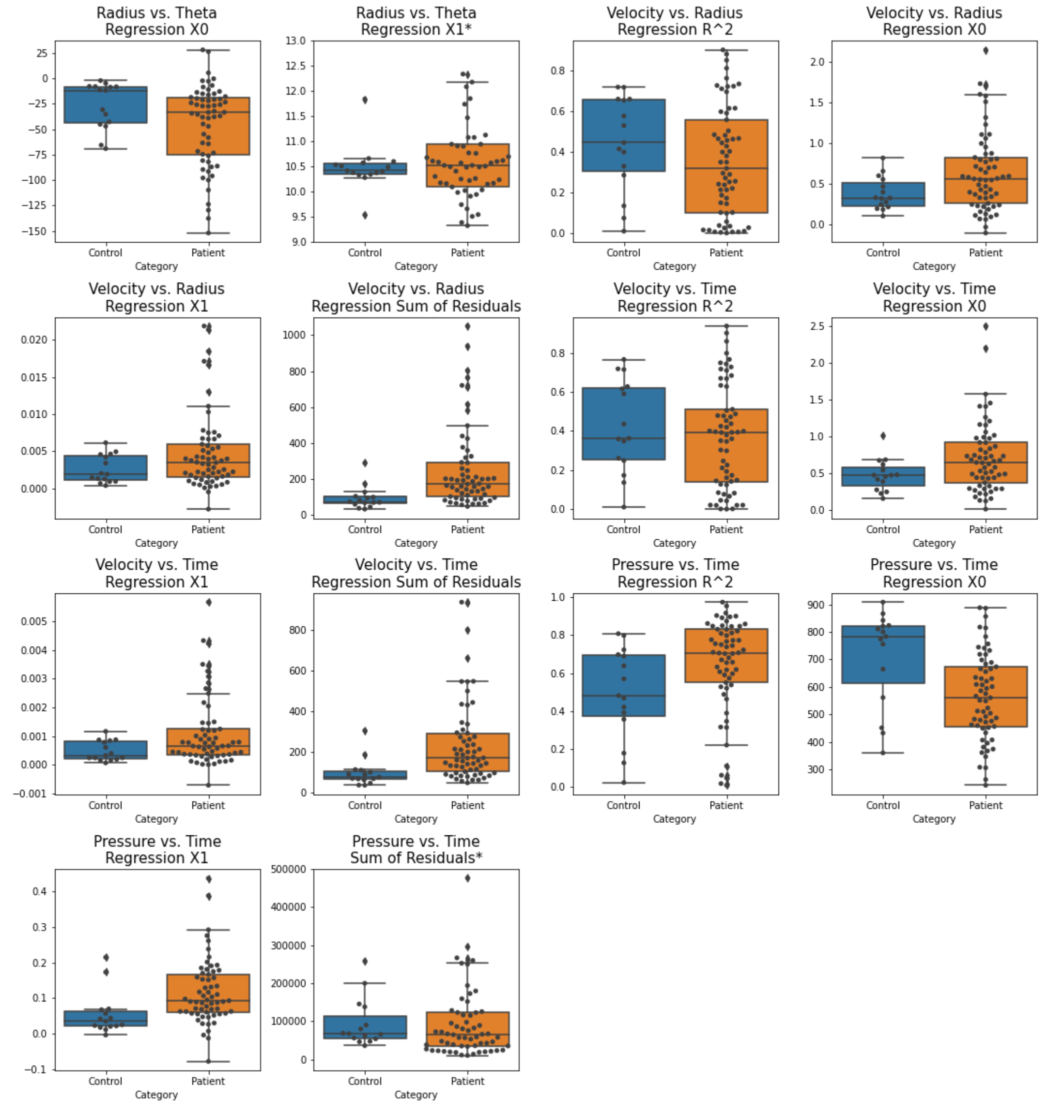

Figure S6. Novel linear regression-based features overlaid box & swarm plots for static spiral tests. The distribution of each linear regression-based feature is visualized for patients and controls separately. Controls are on the left of each sub figure and have a blue box plot. Patients are on the right of each sub figure and have an orange box plot.

Note that for the radius vs. theta linear regression, this figure doesn't include the regression R-squared value and the sum of residuals feature plots, which are already displayed in Figure 2 as some of the most informative features.

Inversely Proportional Relationship between Velocity & Curvature

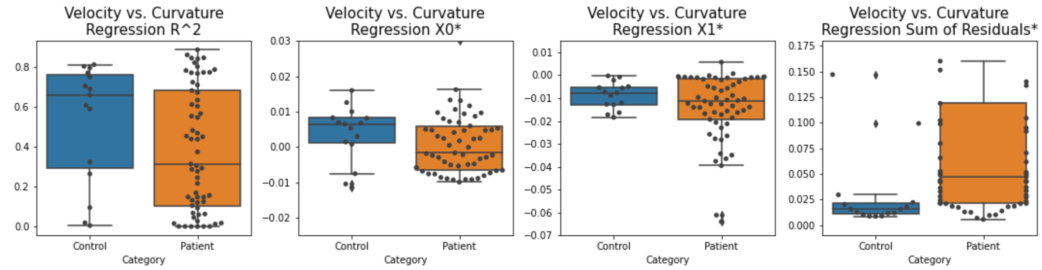

Figure S7. Overlaid swarm & box plots for the novel features based on the inversely proportional relationship between velocity & curvature, for static spiral tests. The distribution of each curvature on velocity regression-based feature is visualized for patients and controls separately. Controls are on the left of each sub figure and have a blue box plot. Patients are on the right of each sub figure and have an orange box plot.

Mean & Standard Deviation of the Rates of Change of Radius and Theta

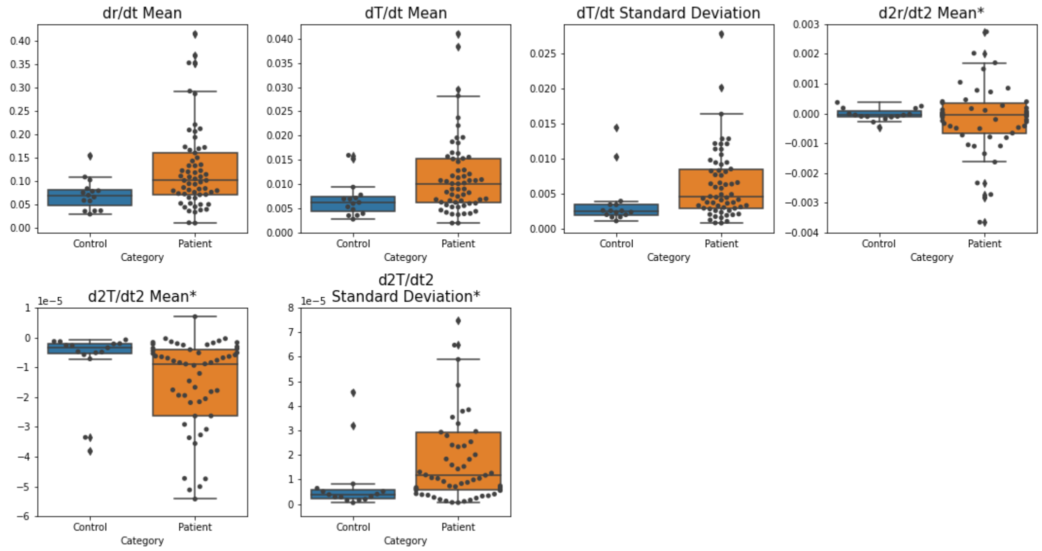

Figure 8. Overlaid swarm & box plots for the novel features based on the rates of change of radius and theta, for static spiral tests. The distribution of each rate of change in radius and theta-based feature is visualized for patients and controls separately. Controls are on the left of each sub figure and have a blue box plot. Patients are on the right of each sub figure and have an orange box plot.

Note that $\frac{d^2r}{dt^2}$ Standard Deviation and $\frac{dr}{dt}$ Standard Deviation feature plots are not included here. They are displayed in Figure 2 as some of the most informative features.

Mean & Standard Deviation of the Rate of Change of Radius with Respect to Theta

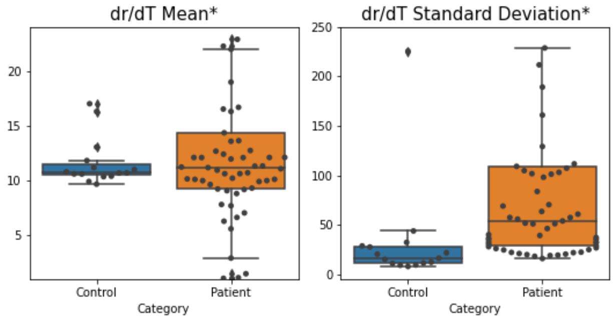

Figure S9. Overlaid swarm & box plots for the novel features based on the rates of change of radius with respect to theta, for static spiral tests. The distribution of the mean and standard deviation of the rate of change in radius with respect to theta is visualized forpatients and controls separately. Controls are on the left of each sub figure and have a blue box plot. Patients are on the right of each sub figure and have an orange box plot.

6. Dynamic Features Visualization

The following series of figures present the overlaid box plots and swarm plots for the newly proposed features that didn't meet the previously mentioned selection criteria for the "most informative features" for the dynamic spiral drawings. In this set of figures, the plots marked with asterisks have sets of outliers excluded to enhance plot visibility.

Curvature-Based Novel Features

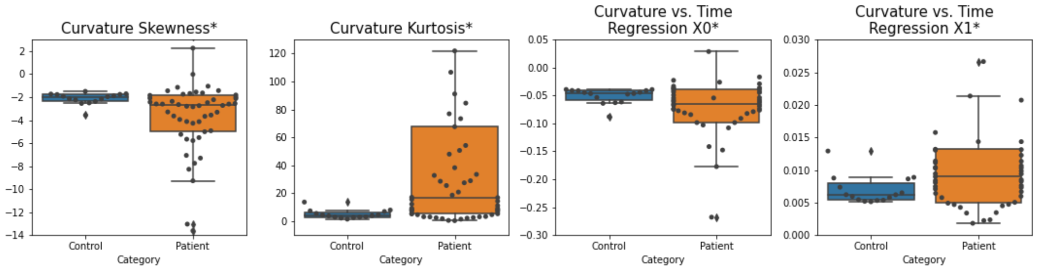

Figure S10. Novel curvature-based features overlaid box & swarm plots, for dynamic spiral tests. The distribution of each curvature feature is visualized for patients and controls separately. Controls are on the left of each sub figure and have a blue box plot. Patients are on the right of each sub figure and have an orange box plot.

"Curvature vs. Time Regression R^2" and "Curvature vs. Time Regression Sum of Residuals" plots aren't included in the figure above and are presented in the most informativedynamic features figure (figure 3).

Fourier Transform-Based Features

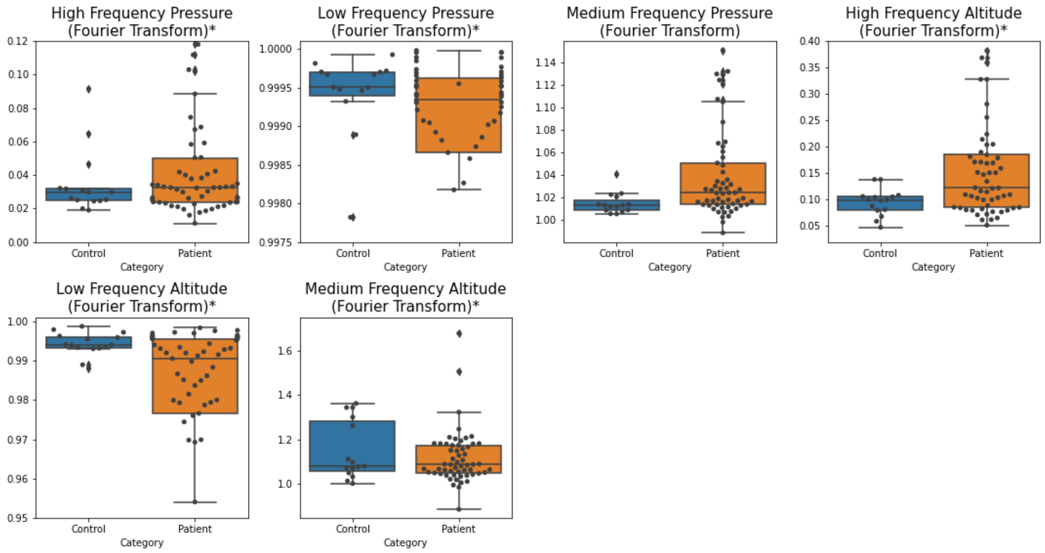

Figure S11. Novel Fourier transform-based features overlaid box & swarm plots for dynamic spiral tests. The distribution of each Fourier transform-based feature is visualized for patients and controls separately. Controls are on the left of each sub figure and have a blue box plot. Patients are on the right of each sub figure and have an orange box plot.

Linear Regression Based Features

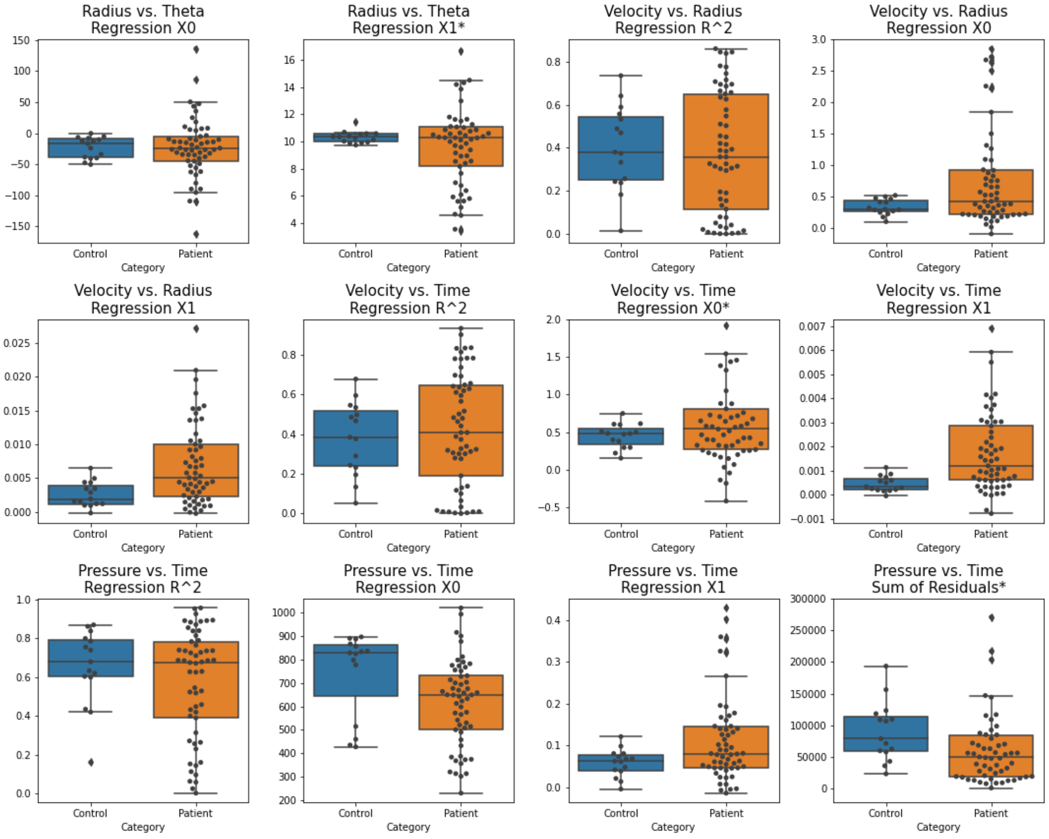

Figure S12. Overlaid box & swarm plots for novel linear regression-based features for dynamic spiral tests. The distribution of each linear regression-based feature is visualized for patients and controls separately. Controls are on the left of each sub figure and have a blue box plot. Patients are on the right of each sub figure and have an orange box plot.

Note that this figure doesn't include "Radius vs. Theta Regression Sum of Residuals", "Radius vs. Theta Regression R^2", "Velocity vs. Radius Regression Sum of Residuals" and "Velocity vs. Time Regression Sum of Residuals" feature plots, which are already displayed inFigure 3 as some of the most informative features.

Inversely Proportional Relationship between Velocity & Curvature

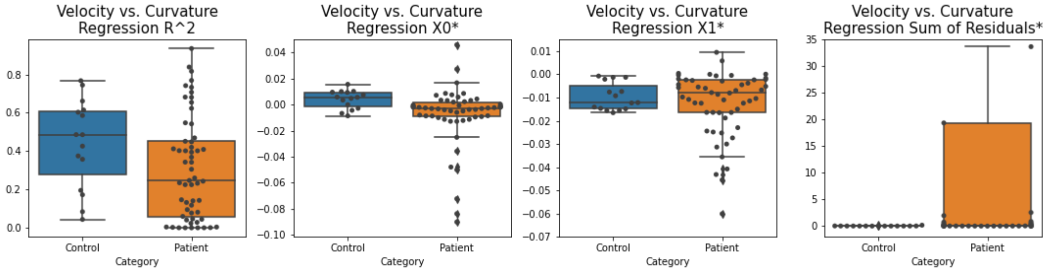

Figure S13. Overlaid swarm & box plots for the novel features based on the inversely proportional relationship between velocity & curvature, for dynamic spiral tests. The distribution of each curvature on velocity regression-based feature is visualized for patients and controls separately. Controls are on the left of each sub figure and have a blue box plot. Patients are on the right of each sub figure and have an orange box plot.

Mean & Standard Deviation of the Rates of Change of Radius and Theta

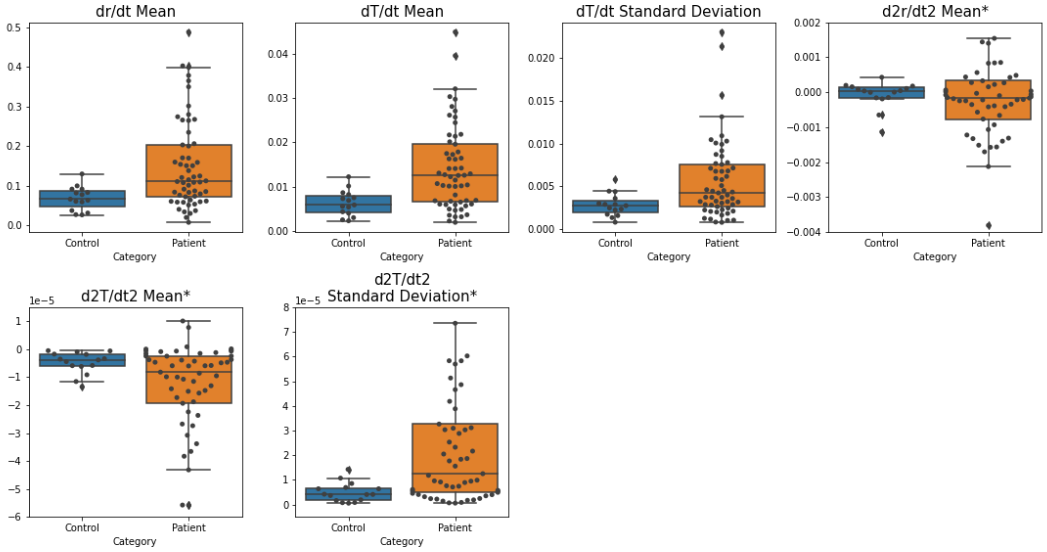

Figure S14. Overlaid swarm & box plots for the novel features based on the rates of change of radius and theta, for dynamic spiral tests. The distribution of each rate of change in radius and theta-based feature is visualized for patients and controls separately. Controls are on the left of each sub figure and have a blue box plot. Patients are on the right of each sub figure and have an orange box plot.

Note that $\frac{d^2r}{dt^2}$ Standard Deviation and $\frac{dr}{dt}$ Standard Deviation feature plots are not included here. They are displayed in Figure 3 as some of the most informative features.

Mean & Standard Deviation of the Rate of Change of Radius with Respect to Theta

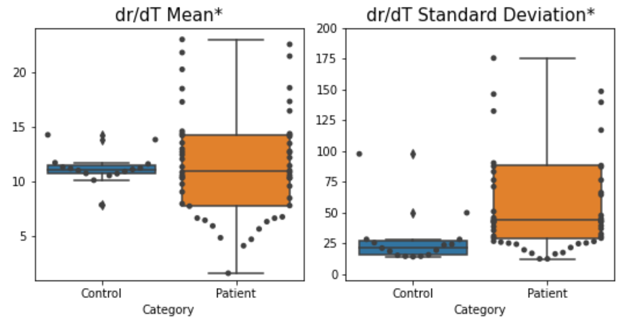

Figure S15. Overlaid swarm & box plots for the novel features based on the rates of change of radius with respect to theta, for dynamic spiral tests. The distribution of the mean and standard deviation of the rate of change in radius with respect to theta is visualizedfor patients and controls separately. Controls are on the left of each sub figure and have a blue box plot. Patients are on the right of each sub figure and have an orange box plot.

7. Explanation of Further Informative Features

The $\frac{d^2r}{dt^2}$ standard deviation, mean jerk, and acceleration standard deviation were other novel features that proved to be very useful in separating patient and control classes. The observed higher values in these metrics may indicate that patients exhibit more periods of sporadic, jerky motion during which the radius of their spiral was increasing at a very fast or very slow rate compared to controls. This could be a result of levodopa-induced dyskinesia [9].

The sum of residuals in regression features such as velocity vs. radius and curvature vs. time was generally observed to be higher in PD patients than controls. The geometry of the Archimedean spiral defines strict mathematical relationships within these pairs of features (e.g. velocity is expected to be proportional to the radius), so deviations indicate atypical completion of the task. Our results indicate that such deviations occurred much more often in PD patients than in controls, possibly due to reduced fine motor control in patients [10].

Within the realm of handwriting and tracing tasks, pressure-based features have been generally important in distinguishing characteristic motor symptoms associated with PD [8,11,12]. In this study, all pressure features were informative under a 0.05 p-value cutoff. Empirically, patients with PD tend to either write with lower absolute pressure (compared to controls) or start off with a typical level of pressure but are unable to maintain it throughout the task [13,8]. Moreover, these distinctive pressure patterns have been associated with well-documented phenomena such as micrographia, bradykinesia, and tremors in PD patients [14]. In sum, quantitative analysis of pressure-based features has been shown to provide diagnostically useful information to discriminate between similar-looking spirals drawn by controls and PD patients.

Table S1. Complete list of features calculated.

Features	Description
Velocity-Based Basic Statistical Measures	Mean, max, standard deviation of velocity
Normal Velocity Variability (NVV)	Characterizes the variability of drawingvelocity
Acceleration-Based Basic StatisticalMeasures	Mean, max, standard deviation of acceleration
Jerk-Based Basic Statistical Measures	Mean, max, standard deviation of jerk
Pressure-Based Basic Statistical Measures	Mean, max, standard deviation of pressure
Entropy of X, Y, velocity, acceleration, jerk, pressure, curvature	Shannon entropy calculations on the listeddata
Skewness of X, Y, velocity, acceleration, jerk, pressure, curvature	Skewness calculations on the listed data
Kurtosis of X, Y, velocity, acceleration, jerk, pressure, curvature	Kurtosis calculations on the listed data
Fourier Transform on Pressure & Pen Altitude	Relative amount of high frequency, medium frequency, and low frequency content in the pressure and pen altitude signal
Regression/Curve Fit-based Features for Pres-sure, Curvature, and Velocity with respectto time	Sum of residuals, R^2, intercept (X0 constant), slope (X1 constant) for each regression/curvefit
Curve Fit-based Features for Curvature with respect to Velocity	Sum of residuals, R^2, intercept (X0 constant), slope (X1 constant)
Rate of Inversions in Velocity, Acceleration,Jerk, Pressure & Curvature	Number of inversions (local maxima) in data / time duration of drawing
Overall Time Duration	Time Spent Drawing (both on-surface &in-air)
Time duration of components of pressuresignal	Time duration of the rising edge and fallingedge of the pressure signal
Numerical range of components of pressure signal	Numerical range of the rising edge and falling edge of the pressure signal
Rate of change in Radius with respect totime-based features	Mean and standard deviation of rate of changein radius
Rate of change in Theta with respect totime-based features	Mean and standard deviation of first order and second order rate of change in theta
Rate of change in Radius with respect toTheta-based features	Mean and standard deviation of first order and second order rate of change in radius with re-spect to theta
Regression-based Features for Velocity with respect to Radius	Sum of residuals, R^2, intercept (X0 constant), slope (X1 constant)
Regression-based Features for Radius withre-spect to Theta	Sum of residuals, R^2, intercept (X0 constant), slope (X1 constant)

References

1. Viviani, P.; Terzuolo, C. Trajectory Determines Movement Dynamics. *Neuroscience* **1982**, *7*, 431–437. https://doi.org/10.1016/0306-4522(82)90277-9.
2. Kotsavasiloglou, C.; Kostikis, N.; Hristu-Varsakelis, D.; Arnaoutoglou, M. Machine Learning-Based Classification of Simple Drawing Movements in Parkinson's Disease. *Biomed. Signal Process. Control* **2017**, *31*, 174–180. https://doi.org/10.1016/j.bspc.2016.08.003.
3. Drotar, P.; Mekyska, J.; Rektorova, I.; Masarova, L.; Smekal, Z.; Faundez-Zanuy, M. Decision Support Framework for Parkinson's Disease Based on Novel Handwriting Markers. IEEE Trans. *Neural Syst. Rehabil. Eng.* **2015**, *23*, 508–516. https://doi.org/10.1109/TNSRE.2014.2359997.
4. Drotár, P.; Mekyska, J.; Rektorová, I.; Masarová, L.; Smékal, Z.; Faundez-Zanuy, M. Analysis of In-Air Movement in Handwriting: A Novel Marker for Parkinson's Disease. *Comput. Methods Programs Biomed.* **2014**, *117*, 405–411. https://doi.org/10.1016/j.cmpb.2014.08.007.
5. Thomas, M.; Lenka, A.; Kumar Pal, P. Handwriting Analysis in Parkinson's Disease: Current Status and Future Directions. *Mov. Disord. Clin. Pract.* **2017**, *4*, 806–818. https://doi.org/10.1002/mdc3.12552.
6. Loconsole, C.; Trotta, G.F.; Brunetti, A.; Trotta, J.; Schiavone, A.; Tatò, S.I.; Losavio, G.; Bevilacqua, V. Computer Vision and EMG-Based Handwriting Analysis for Classification in Parkinson's Disease. In *Intelligent Computing Theories and Application*; Huang, D.-S., Jo, K.-H., Figueroa-García, J.C., Eds.; Lecture Notes in Computer Science; Springer International Publishing: Cham, Switzerland, 2017; Volume 10362, pp. 493–503. https://doi.org/10.1007/978-3-319-63312-1_43.

7. Schröter, A.; Mergl, R.; Bürger, K.; Hampel, H.; Möller, H.-J.; Hegerl, U. Kinematic Analysis of Handwriting Movements in Patients with Alzheimer's Disease, Mild Cognitive Impairment, Depression and Healthy Subjects. *Dement. Geriatr. Cogn. Disord.* **2003**, *15*, 132–142. https://doi.org/10.1159/000068484.
8. Drotár, P.; Mekyska, J.; Rektorová, I.; Masarová, L.; Smékal, Z.; Faundez-Zanuy, M. Evaluation of Handwriting Kinematics and Pressure for Differential Diagnosis of Parkinson's Disease. *Artif. Intell. Med.* **2016**, *67*, 39–46. https://doi.org/10.1016/j.artmed.2016.01.004.
9. Thanvi, B.; Lo, N.; Robinson, T. Levodopa-Induced Dyskinesia in Parkinson's Disease: Clinical Features, Pathogenesis, Prevention and Treatment. *Postgrad. Med. J.* **2007**, *83*, 384–388. https://doi.org/10.1136/pgmj.2006.054759.
10. Dan, X.; Liu, J.; Doyon, J.; Zhou, Y.; Ma, J.; Chan, P. Impaired Fine Motor Function of the Asymptomatic Hand in Unilateral Parkinson's Disease. *Front. Aging Neurosci.* **2019**, *11*, 266. https://doi.org/10.3389/fnagi.2019.00266.
11. Zham, P.; Kumar, D.K.; Dabnichki, P.; Poosapadi Arjunan, S.; Raghav, S. Distinguishing Different Stages of Parkinson's Disease Using Composite Index of Speed and Pen-Pressure of Sketching a Spiral. *Front. Neurol.* **2017**, *8*, 435. https://doi.org/10.3389/fneur.2017.00435.
12. Zham, P.; Raghav, S.; Kempster, P.; Poosapadi Arjunan, S.; Wong, K.; Nagao, K.J.; Kumar, D.K. A Kinematic Study of Progressive Micrographia in Parkinson's Disease. *Front. Neurol.* **2019**, *10*, 403. https://doi.org/10.3389/fneur.2019.00403.
13. Rosenblum, S.; Samuel, M.; Zlotnik, S.; Erikh, I.; Schlesinger, I. Handwriting as an Objective Tool for Parkinson's Disease Diagnosis. *J. Neurol.* **2013**, *260*, 2357–2361. https://doi.org/10.1007/s00415-013-6996-x.
14. Moustafa, A.A.; Chakravarthy, S.; Phillips, J.R.; Gupta, A.; Keri, S.; Polner, B.; Frank, M.J.; Jahanshahi, M. Motor Symptoms in Parkinson's Disease: A Unified Framework. *Neurosci. Biobehav. Rev.* **2016**, *68*, 727–740. https://doi.org/10.1016/j.neubiorev.2016.07.010.
